# When Love Meets Money: Priming the Possession of Money Influences Mating Strategies

**DOI:** 10.3389/fpsyg.2016.00387

**Published:** 2016-03-21

**Authors:** Yi Ming Li, Jian Li, Darius K.-S. Chan, Bo Zhang

**Affiliations:** ^1^Institute of Developmental Psychology, School of Psychology, Beijing Normal UniversityBeijing, China; ^2^Beijing Key Laboratory of Applied Experimental Psychology, School of Psychology, Beijing Normal UniversityBeijing, China; ^3^Department of Psychology, The Chinese University of Hong KongHong Kong, China

**Keywords:** money, mating strategy, long term, short term, romantic relationship

## Abstract

Money is an important factor that influences the development of romantic relationships. The current paper examines how the feeling of having relatively more or less money influences human mating strategies in long-term and short-term mating contexts under the framework of evolutionary psychology. We recruited mainland Chinese college students involved in steady, heterosexual romantic relationships to participate in two experiments. In each study, we experimentally triggered participants' feelings of having relatively more or less money and then examined their thoughts and behaviors related to mating. Results of Study 1 showed that men who were primed to feel that they had relatively more money were less satisfied with their partners' physical attractiveness than those primed to feel that they had less money, suggesting that the subjective feeling of having more or less money may affect men's preferences regarding the physical appearance of a mate in a long-term relationship. Interestingly, this difference was not significant for women. Results of Study 2 indicated that both men and women who were primed to feel that they had relatively more money exhibited a greater “behavioral approach tendency” toward an attractive member of the opposite sex than those primed to feel that they had less money. This finding suggests that people who feel they have relatively more money may have more interest in an attractive alternative than those who feel they have relatively less money. The differences in mating strategies between and within the genders brought about by money support the evolutionary hypothesis that individuals adopt conditional mating strategies in response to environmental conditions. Additionally, the results of experimental studies provide evidence for the causal effects of money on mating strategies. These findings have both conceptual and practical implications for the psychology of evolution and romantic relationships.

## Introduction

Money is often involved in love stories. There are complex outcomes derived from the meeting of money and romantic love, and it is often difficult to conclude that money is either a promoter or an inhibitor of love. Some scholars have made efforts to explore the relation between money and romantic relationships, with a particular focus on the effects of women's income on the stability of long-term relationships (e.g., Oppenheimer, 1997; Heckert et al., 1998; Rogers, 2004). However, the mixed findings from these studies (e.g., Conger et al., 1990; Rogers, 2004; Teachman, 2010) are not able to adequately explain whether and how money influences the development of romantic relationships. Some researchers have examined the influences of money on human relationships in laboratory experiments (e.g., Vohs et al., 2006; Mogilner, 2010). The experimental methods help to control for the influence of certain factors and allow researchers to make causal conclusions about the effects of money. Researchers have found that money tends to separate people from others and weaken social bonds due to the self-sufficient mental state it creates (Vohs et al., 2006; Mogilner, 2010). However, little is known about whether money can generate the social distancing effect in the mating context. One way to find the answer is to look back to our ancestral past and explore the roles of resources in our ancestors' mating process.

On the basis of previous research findings and techniques, the current studies aimed to explore the effects of money on romantic relationships under the framework of evolutionary psychology. Specifically, we conducted two experiments and examined individuals' mating preferences and selectivity in response to the amount of money they possess in the long-term and extra-pair mating contexts. Attitudes toward a long-term partner and an attractive alternative can well predict the stability and development of relationships (Rusbult, 1983). Therefore, the current paper could provide new evidence on the influence of money on romantic relationships. Additionally, findings from the two studies will help us better understand the processes of human mating and enrich the literature on evolutionary psychology. Next, we review the related literature and elaborate on how we built our research on prior work.

Human mating strategies are solutions to the adaptive problems that our ancestors confronted to achieve reproductive success over our long history. According to Trivers' (1972) theory of parental investment, women devote much more effort and time to offspring than men due to internal gestation, lactation, and extended parental care. Sex differences in terms of minimum parental investment might lead to sex differences in the adaptive problems and optimal strategies used for reproductive success. Specifically, men's reproductive success is constrained by the number of fertile women, whereas women's reproductive success is constrained by their access to the continuous resources from one or more mates to rear children and the quality of a mate's genes. Thus, two significant differences in mating strategies have evolved between men and women. First, men and women differ in the relative importance they place on a mate's physical appearance and resources. Whereas both men and women prefer an attractive mate, men are more likely to value a mate's physical attractiveness, which signals a woman's fertility and reproductive value, than women. On the other hand, women are more likely to attach importance to a mate's resources than men (e.g., Li et al., 2002; Shackelford et al., 2005). Second, men have evolved a stronger interest in short-term mating and desire more mates than women (e.g., Clark and Hatfield, 1989; Buss and Schmitt, 1993).

Massive within-sex differences in mating strategies exist together with these sex differences. For example, physically attractive men are more likely to engage in short-term mating than unattractive men (Lukaszewski et al., 2014). When their own access to independent resources increases, women may place more value on men's physical attractiveness that may signal pathogen-resistant “good genes,” leading them to engage in short-term mating (Gangestad and Simpson, 2000). This flexibility of human mating is well explained by the theory of strategic pluralism (Gangestad and Simpson, 2000). Put simply, both men and women adopt conditional mating strategies depending on specific environmental factors or personal characteristics and aim to maximize or optimize their reproductive opportunities. Next, we focus on material resources or money and discuss how they influence human mating strategies.

The resources acquisition characteristic reflects a man's mate value (Waynforth and Dunbar, 1995; Li et al., 2002; Shackelford et al., 2005). Evolutionary psychologists believe that individuals with high mate value are more likely to choose a sex-typical preferred strategy to achieve reproductive success than those with low mate value (Buss and Schmitt, 1993). Therefore, men with more money can make higher demands with regard to women's physical attractiveness and engage more in short-term mating than men with less money. Results of empirical studies have supported this proposition. For example, Waynforth and Dunbar (1995) used personal advertisement data and found that men offering resources had higher mate standards than those who did not. Yong and Li (2012) found that men had higher requirements for a potential mate when primed with large resources. Furthermore, some researchers suggest that showing immediate resources is an effective way to obtain short-term mating opportunities (Cloyd, 1976; Hill et al., 1987).

According to Chang et al. (2011a) opinion, men engage in wars for acquiring resources and territories, which can enhance their success in male intrasexual competition for female's sexual opportunities. Following this reasoning, possession of money could also be viewed as a cultural extension of weapon-like male attributes which help them win in intrasexual completion. Previous studies have showed that individual differences in weapon-like characteristics (e.g., aggressive personality) were positively associated with intrasexual mating strategies (Chen and Chang, 2015). Thus, cues to resources in the environment may lead men to choose the adaptive strategies to deal with intrasexual competition and maximize their reproductive success. On one hand, when men do not have enough resources such as money to win in intrasexual competition, it would be more beneficial for them to remain stable in the current relationship. Thus, men with less money may set lower standards for the current partner and tend to be more satisfied with her than men with more money. On the other hand, men with more money may become more confident and dominant in intrasexual competition, and are more likely to maximize their reproductive success by seeking more mates.

Generally, physical attractiveness reflects a woman's mate value more than material resources do (Koyama et al., 2004; Todd et al., 2007). Historically, material resources or money are directly related to women's primary adaptive problem, so the amount of money that women possess might affect their reproductive benefit-cost analysis. According to strategic pluralism theory (Gangestad and Simpson, 2000), women make a trade-off between a mate's parental investment and genetic benefits contingent on environmental conditions. When women possess sufficient resources to rear children independently, they may have less need for men's resources and benefit more from choosing a partner with good heritable qualities. In this case, women may place more value on the physical appearance of a long-term mate and/or engage in a short-term relationship with an attractive mate.

Some studies have provided evidence that women's access to resources is indeed associated with an increased mate preference for physical attractiveness (e.g., Gangestad, 1993; Koyama et al., 2004). However, in the experiment conducted by Yong and Li (2012), women did not increase their mate standards for physical attractiveness when primed with a larger sum of money. We believe that differences in factors such as research methodology, selection of variable indicators, and sample characteristics among these studies might contribute to the mixed findings. For example, in some previous studies, participants who were not required to be involved in a romantic relationship were instructed to give preference ratings on a potential or imagined partner (e.g., Koyama et al., 2004). If women are already committed to a long-term relationship, they might not get more reproductive output by making higher demands regarding the unchangeable physical characteristics of a current partner. In this regard, an increased mating standard for physical attractiveness may impair the stability of the current relationship. Losing a long-term relationship has a larger reproductive cost for women than for men. Therefore, historically, relationship status of women could influence their mating strategies. Even if committed women possess sufficient resources, they might not increase their demands with regard to a long-term mate's physical appearance.

On the other hand, researchers have highlighted the possibility that women seek good genes through extra-pair mating (Pillsworth and Haselton, 2006). They believe that sometimes it would be adaptive for some women to secure sufficient resources from a long-term partner and obtain heritable benefits from extra-pair mates. Similarly, it is reasonable to believe that women are more likely to engage in extra-pair mating when they have their own access to money and depend less on men's resources. However, previous studies indicated that women are more likely to protect the relationships than men (e.g., Cross et al., 2000; Lydon et al., 2008). Thus, we expect that the amount of money on possesses would cause a smaller variance in women's extra-pair mating than in men's.

As mentioned earlier, women have less need for men's resources when they possess their own resources to take care of children. Even so, they may not reduce their mating standard for resources. Theoretically, they can maintain the mating standard that matches with their physical attractiveness (Pawlowski and Dunbar, 1999) or increase it due to the positive assortative mating effect (Kalmijn, 1994). The structural powerlessness hypothesis (Buss and Barnes, 1986) provides another possibility. According to this hypothesis, women are excluded from power and so they get resources by seeking for a mate with power and status. Consequently, when men and women are endowed with equal power and resources, women would reduce their demands for a mate's resources and the differences between the sexes in terms of mate preferences would decrease.

Previous studies show that attitudes related to sexual equality are indeed associated with a decrease in women's preferences regarding a mate's financial status (Eagly and Wood, 1999; Moore et al., 2006). However, access to or possession of resources may not be associated with women's decreased demand for a mate's resources (Buss, 1989; Gil-Burmann et al., 2002; Yong and Li, 2012). Empirical evidence reveals that power-related sexual equality and money may exert different influences on human mating strategies. The structural powerlessness hypothesis does not seem to be suitable to explain money's effect on women's preference for a mate with resources. Instead, these research findings supported the evolutionary proposition that women value men's resources regardless of their own possession of wealth.

Taken together, the findings show that money is an important factor leading to differences in mating strategies within each sex. Specifically, both men and women who have more money are more likely to attach more importance to a mate's physical attractiveness and to engage in short-term mating than those who have less money. However, for committed women, money may lead to less variation in their mating strategies. These propositions are based on evolutionary theory and research, but most of the related studies used a correlational design. Therefore, empirical evidence generated from experimental research is needed to establish the causal effects of money on mating strategies.

The purpose of the current research is to examine the causal effects of the feeling of having relatively more or less money on human mating strategies. We are particularly interested in sex differences and within-sex variations in this effect. Specifically, we arranged long-term and extra-pair mating contexts in two experiments, respectively. In Study 1, we examined individuals' satisfaction with their current partners so that we could determine the difference in preferences for a long-term mate between individuals primed to feel that they had relatively more money and individuals primed to feel that they had relatively less money. In Study 2, we assessed individuals' behavioral response to an attractive potential extra-pair mate based on money priming.

It should be noted that the participants were college students who were involved in an exclusive dating relationship and expected the relationship to last for more than 10 years. It is reasonable to believe that these dating couples had a long-term relationship plan and had selected each other as a long-term mate. Therefore, we considered the participants to be in a long-term mating context, and their encounter with an attractive alternative could be interpreted as short-term, opportunistic mating. Additionally, instead of using income as an indicator of money possession, we experimentally manipulated individuals' subjective feeling of having relatively more or less money. We believed that a subjective feeling regarding wealth could be a more direct influence on romantic relationships than the actual amount of money because psychological evaluations of monetary income could be different for different people.

## Study 1

Study 1 examined whether and how the feeling of having relatively more or less money would influence individuals' satisfaction with their current partners in a long-term relationship. Satisfaction with a romantic partner was measured with regard to physical attractiveness and resources. Our major hypothesis is as follows:

Men, not women, who feel they have relatively more money would be less satisfied with their current partners' physical attractiveness than those who feel they have relatively less money. That is, a gender by money priming interaction on participants' satisfaction with their partners' physical attractiveness would be significant. However, such a gender by money priming interaction would not be observed for participants' satisfaction ratings on their partners' resources.

### Methods

#### Participants

A total of 182 undergraduate and postgraduate students (121 women, 61 men), primarily from universities in Beijing, China, participated in this study. Their ages ranged from 18 to 27, with a mean of 20.91 (*SD* = 1.81). All of the participants were heterosexual and involved in a dating relationship during the survey period. The length of their ongoing relationships ranged from 2 months to 7 years, with a mean of 20.28 months (*SD* = 15.95). Their monthly incomes (mainly from their family or/and part-time jobs) varied from 400 to 5000 RMB, with a mean of 1472.58 RMB (*SD* = 833.25). The study is approved by the Ethics Board of School of Psychology, Beijing Normal University.

#### Procedure

We employed the money-priming method used by Nelson and Morrison (2005) to induce the relatively rich or poor feeling. Participants were randomly assigned to the relatively wealthy or relatively poor condition and were asked to respond to some questions about financial status. The response scale was in fact different between the two conditions. For example, one question was about the amount of money in their savings accounts: Participants in the relatively wealthy condition provided ratings on a 7-point scale divided into much smaller increments (i.e., from 1 [RMB0–RMB250] to 7 [over RMB500]) than those in the relatively poor condition (i.e., from 1 [RMB0–RMB2000] to 7 [over RMB12000]). We expected that most of the participants in the relatively wealthy condition would choose the highest amount of money and that those in the relatively poor condition would choose the bottom of the scale. Participants receiving such a money-priming manipulation generally believe that the scale is constructed on the basis of the distribution of the actual income of college students and that the top of the scale reflects the highest level of income and the bottom reflects the lowest (Schwarz, 1999). Therefore, we expected that the relatively wealthy group would be relatively satisfied with their personal financial status, whereas the relatively poor group would be less satisfied. After finishing this money rating, participants were asked to indicate whether they were satisfied with their personal finances using a 9-point Likert scale (1 = not at all satisfied, 9 = very satisfied) as a manipulation check.

Following the money primes, participants were asked to complete a measure of satisfaction with their romantic partners and to answer demographic questions about gender, age, and monthly income.

### Materials

#### Satisfaction with a romantic partner

The scale of satisfaction with a romantic partner consisted of two dimensions, physical attractiveness and resources, which were adapted from the short version of Fletcher et al.'s (1999) ideal partner scales. Due to time constraints, we shortened the scale by selecting four items in each dimension with the highest item-total correlations. Some of the items were modified because they were unsuitable for college students. For instance, we changed the item “good job” to “good job prospects.” We also replaced the item “extraverted” with “good looking” because “extraverted” is not an attractive attribute that Chinese men value (Wang et al., 2015) and we tried to make the items in this dimension center on physical appearance. Participants rated their partner's physical attractiveness (attractive, nice body, good looking, sexy) and resources (good job prospects, financially secure, good family background, successful) on a 9-point Likert scale (1 = does not match my ideal at all, 9 = completely matches my ideal). A higher score suggested that the current partner matched the ideal partner more closely and that the individual was more satisfied with his/her partner. The reliability coefficient was 0.88 for the dimension of physical attractiveness and 0.84 for the dimension of resources.

### Results and discussion

The first set of results is on the manipulation check, which examines whether the money priming method is effective. The second set presents descriptive statistics of the study variables. In the next sections, ANCOVAs are employed to examine whether the feeling of having relatively more or less money affects an individual's satisfaction with his/her partner.

#### Manipulation check

The result of a *t*-test showed that participants in the relatively wealthy condition (*M* = 6.20, *SD* = 1.42) did feel more satisfied with their financial status than those in the relatively poor condition [*M* = 5.25, *SD* = 1.65; *t*_(180)_ = 4.14, *p* < 0.001, Cohen's *d* = 0.61]. This finding suggested that the money-priming method was successful.

#### Descriptive analysis

The means and standard deviations of the dependent variables by experimental condition and gender are presented in Table 1.

**Table 1:** **Means and standard deviations of the dependent variables by gender and experimental condition for Study 1 and Study 2**.

		**Male**		**Female**		**Total sample**	
		***M***	***SD***	***M***	***SD***	***M***	***SD***
Study 1	**Physical**						
	**attractiveness**						
	Relatively wealthy	6.01	1.66	6.12	1.33	6.08	1.45
	Relatively poor	7.00	1.17	6.15	1.37	6.42	1.36
	**Resources**						
	Relatively wealthy	5.83	1.56	5.97	1.26	5.92	1.37
	Relatively poor	6.14	1.28	6.18	1.32	6.17	1.30
Study 2	**Distance**						
	Relatively wealthy	1.89	0.67	2.50	1.25	2.13	0.98
	Relatively poor	2.38	1.01	2.75	1.23	2.52	1.11

*Scale ranges from 1 to 9 for satisfaction with a partner's physical attractiveness and resources and from 1 to 5 for distance*.

#### Satisfaction with partner's physical attractiveness

Given the possible influences of actual income (Rogers, 2004) on relationship outcomes, we controlled for its effect on the dependent variables statistically. Specifically, we used an ANCOVA to examine the influence of the subjective feeling of the amount of money one possesses on individuals' satisfaction with their partners' physical attractiveness after controlling for the potential confounding effects of actual income on the dependent variable. Money-priming condition and participant gender served as between-subject factors. As predicted, there was a significant interaction between money-priming condition and participant gender, *F*_(1, 177)_ = 5.07, *p* = 0.026, η^2^_*partial*_ = 0.028, suggesting that gender significantly moderated the influence of the feeling of having relatively more or less money on individuals' satisfaction with their partners' physical attractiveness.

**Figure 1:**
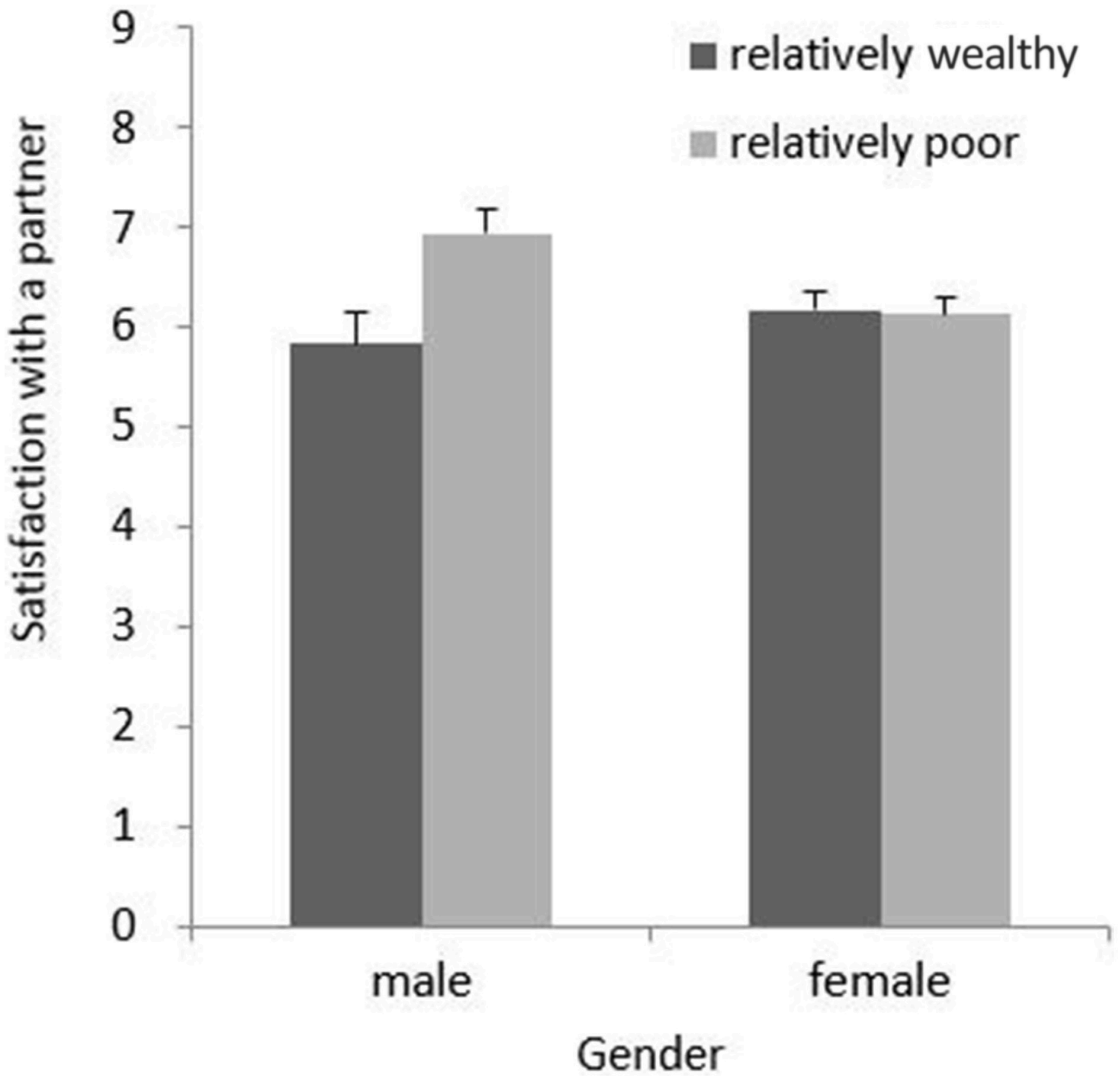
**Simple effect analysis demonstrating the moderating effect of gender on the influence of the feeling of having relatively more or less money on satisfaction with a partner's physical attractiveness**. Actual income was included in the analysis as a covariate. Error bars represent standard errors of the mean.

We used the Bonferroni method to conduct the simple effect analysis and adjusted the alpha level (α = 0.05/2 for two comparisons) to control for Type I errors. Consistent with our major hypothesis, the main effect of money-priming condition was significant for men, *F*_(1, 177)_ = 6.68, *p* = 0.011, η^2^_*partial*_ = 0.036. The men in the relatively wealthy condition (*M* = 6.01, *SD* = 1.66) were less satisfied with their partners' physical appearance than those in the relatively poor condition (*M* = 7.00, *SD* = 1.17). For the women, the effect of the money-priming condition was not significant, *F*_(1, 177)_ = 0.11, *p* = 0.746. The interaction pattern is depicted in Figure 1.

#### Satisfaction with partner's resources

Similarly, an ANCOVA was used to examine the influence of the subjective feeling of the amount of money one possesses on individuals' satisfaction with their partners' resources after controlling for the potentially confounding effects of actual income on the dependent variable. The results showed that none of the main variables, gender [*F*_(1, 177)_ = 0.544, *p* = 0.462], money condition [*F*_(1, 177)_ = 0.541, *p* = 0.463], or their interaction term [*F*_(1, 177)_ = 0.06, *p* = 0.807], had a significant effect. This suggests that, for both men and women, the feeling of having relatively more or less money does not affect individuals' satisfaction with partners' resources.

In summary, results of Study 1 supported our major hypothesis. The men who subjectively felt that they had relatively more money perceived a greater discrepancy between their ideal and their current partners in terms of physical appearance and were less satisfied with their partners than those who felt they had less money, but this effect did not occur in women. This result is consistent with Yong and Li's (2012) finding that men increase their requirement for a potential partner's physical attractiveness when primed with larger resources, while with the same resources prime, women do not change their standards.

## Study 2

In Study 2, we examined whether priming the possession of money would influence the use of specific mating strategies in an extra-pair mating context in which committed participants were led to believe that they would have an encounter with an attractive person of the opposite sex. In this study, we used a mental simulation method to prime the feeling of having relatively more or less money. This mental simulation procedure has been frequently employed to generate a psychological state. For example, Vohs et al. (2006) asked participants to read an essay about growing up having either abundant financial resources or meager resources; the procedure successfully activated the idea of having an abundant or a restricted amount of money. Slightly different from previous studies, we provided an incomplete essay and asked participants to fill in the blanks by using their imagination on the computer. We expected that the fill-in-the-blanks task would lead the participants to engage in deeper cognitive processing leading to the creation of self-related information rather than just reading (Craik and Lockhart, 1972), thus effectively generating the relatively rich or poor feelings.

We set up an extra-pair mating situation by arranging a fictitious encounter with an attractive member of the opposite sex. We examined whether the feeling of having relatively more or less money would change the tendency of the dating individuals to approach the attractive alternative. This tendency to approach may reflect the likelihood of the individuals using the extra-pair mating strategy. Similar to Lydon et al.'s (2008) study, we measured individuals' tendency to approach an attractive alternative mate by the distance they chose to sit away from this attractive person in a subsequent interaction; a shorter distance indicated a stronger tendency to approach the attractive alternative. We expected that this behavioral measure would exclude the interference of social desirability and induce a more realistic and genuine response (Fazio and Olson, 2003). We also measured the emotional state of the participants at the end of the experiment to exclude the possibility that the observed effects were caused by immediate emotion. In summary, two hypotheses were proposed:

H1: Individuals who feel they have relatively more money would sit closer to an attractive alternative than those who feel they have relatively less money.

H2: Gender would moderate the relation between the feeling of having relatively more or less money and the distance in such a way that the effect of the feeling on the distance would be stronger for men than for women.

### Methods

#### Participants

In this study, the participants were 121 undergraduate and postgraduate students (48 women, 73 men) primarily from universities in Beijing, China. Their ages ranged from 18 to 30, with a mean of 22.91 (*SD* = 2.34). All of the participants were heterosexual and involved in a dating relationship during the survey period. The length of their ongoing relationships ranged from 2 months to 8 years, with a mean of 26.37 months (*SD* = 21.47). Their monthly income varied from 600 to 8000 RMB, with a mean of 1590.60 RMB (*SD* = 1152.66). The study is approved by the Ethics Board of School of Psychology, Beijing Normal University.

#### Procedure

We performed a pilot study to examine the effectiveness of the money prime before the experiment. The feeling of having relatively more or less money was triggered by asking participant to imagine being in a rich or poor situation and filling in some blanks to complete a story. Specifically, 56 participants (17 men, 37 women) were randomly assigned to two manipulations. In the relatively wealthy condition, participants were asked to imagine how they would live a luxurious life after winning the lottery; they completed sentences such as “*I bought myself*.” Participants in the relatively poor condition were asked to imagine how they would live a miserable life after losing a huge sum of money; they completed sentences such as “*First, about food*.” To determine the effectiveness of this money priming technique, participants were asked to rate how relatively wealthy they felt on a 9-point Likert scale. Result of a *t*-test showed that participants in the relatively wealthy condition (*M* = 4.83, *SD* = 1.56) did feel relatively wealthier than those in the relatively poor condition [*M* = 4.04, *SD* = 1.25; *t*_(54)_ = 2.09, *p* = 0.042, Cohen's *d* = 0.56]. This suggests that the money priming method is valid.

A few days before the actual experiment, the participants provided demographic information, including age, gender, relationship status, and monthly income. Upon arrival at the laboratory, they were told that the experiment consisted of an imagination test and an investigation related to social perception aiming to make a comparison between an impression formed by looking at a photograph and an impression formed by a face-to-face interaction. The arrangement of a face-to-face interaction was used to make participants believe that there was an opportunity to encounter an attractive member of the opposite sex, but this did not actually happen.

After the participants finished imagining a rich or poor life, we showed them a photograph of an attractive person of the opposite sex. Before the experiment, we asked 10 men and 10 women to rate four photographs of an attractive person of the opposite sex on a 9-point Likert scale (1 = completely unattractive, 9 = very attractive). We then selected the two photos (one male, one female) that received the highest attractiveness ratings with the least variance (*M*_*male*_ = 6.92 *SD* = 0.90; *M*_*female*_ = 7.22, *SD* = 1.30). Participants were told to evaluate this opposite-sex individual by his/her photograph and that they would then have a 3-min face-to-face conversation with him/her. After the evaluation, participants were led to the next room, which had a long desk and six chairs. For half of the participants, a bag, a coat, and a book occupied the position closest to the door at one end of the group of chairs, while for the other half of the participants, these items were placed at the position furthest from the door at the other end of the group of chairs. Thus, we controlled for the influence of distance from this position to the door on the participants' choices. Participants were told that the person they would be talking to had been sitting on the seat with the items and would come back soon. They were asked to take a seat and wait for a moment. They had five choices of chair (from 1 = “closest to” to 5 = “furthest from” this fictitious other's seat). Their chair choice represented their chosen distance from the attractive alternative. When they sat down, the experimenter recorded their choice and gave them the PANAS Scale (Watson et al., 1988) to complete.

We then assessed whether the participants were suspicious of the cover story. Three participants were removed from the subsequent analyses because of their suspicions. Finally, we debriefed the participants.

### Materials

#### PANAS

The 20-item Positive and Negative Affect Schedule (Watson et al., 1988) was used to measure the emotional state of participants in the experiment. PANAS consists of a positive affect scale and a negative affect scale. The participants were asked to rate each positive or negative affect on a 5-point scale ranging from “not at all” to “extremely.” In the current study, the reliability coefficient was 0.86 for the positive affect scale and 0.77 for the negative affect scale.

### Results and discussion

Three sets of results are presented below. First, descriptive statistics of the study variables are presented. Second, an ANCOVA is employed to examine the effect of money priming on the distance the participants chose to sit away from the attractive alternative and the moderating effect of gender. In the third section, we present findings on whether emotion might have influenced the participants' choice of seat.

#### Descriptive analysis

The means and standard deviations of the main variables by experimental condition and gender are presented in Table 1.

#### Distance from the participants to the attractive alternative

We conducted an ANCOVA to test the influence of the feeling of having more or less money on the distance the participants chose to sit away from the attractive alternative and the moderating effect of gender. Actual income and the bag's position in the experimental arrangement were considered covariates. Money-priming condition and participant gender served as between-subjects factors.

Results indicated that the interaction between money-priming condition and gender was not significant, *F*_(1, 115)_ = 0.21, *p* = 0.651, suggesting that gender was not a significant moderator. Both money condition [*F*_(1, 115)_ = 4.42, *p* = 0.038,η^2^_*partial*_ = 0.037] and gender [*F*_(1, 115)_ = 9.81, *p* = 002, η^2^_*partial*_ = 0.079] had significant effects on how close participants chose to sit to the attractive alternative's seat. That is, individuals in the relatively wealthy condition (*M* = 2.13, *SD* = 0.98) selected a closer seat to the attractive alternative than those in the relatively poor condition (*M* = 2.52, *SD* = 1.11). In addition, the men (*M* = 2.14, *SD* = 0.89) chose a closer seat than the women (*M* = 2.63, *SD* = 1.23). Thus, the feeling of having relatively more money motivates individuals to approach attractive alternatives more closely than the feeling of having relatively less money does. In other words, individuals who feel they have relatively more money seem to be more likely to use the extra-pair mating strategy than those who feel they have relatively less money.

#### Emotion

To test whether the differences in the tendency to approach an attractive alternative were caused by emotion, we conducted a *t*-test to compare the differences in positive mood and negative mood across conditions. We did not find any significant variation between the relatively wealthy and relatively poor conditions in either mood valence [*t*_(119)positive_ = −0.1, *p* = 0.921; *t*_(119)negative_ = 0.02, *p* = 0.982]. Next, we followed Dienes' procedure (2014) and calculated a Bayes factor to check whether the differences of mood between the two conditions were really nonsignificant. PANAS is a 1–5 likert scale and the difference between conditions cannot be more than four. We used the Dienes (2008) Bayes factor calculator (http://www.lifesci.sussex.ac.uk/home/Zoltan_Dienes/inference/bayes_factor.swf) and assumed a uniform distribution. We entered “0” as the lower bound and “4” as the upper bound. For positive mood, the sample mean was −0.01186, and the standard error was 0.11874. Results showed that the likelihood of the data given the theory was 0.1142, the likelihood of the data given the null was 3.3431, and the Bayes factor was 0.03. For negative mood, the sample mean was 0.00137, and the standard error was 0.05957. Results showed that the likelihood of the data given the theory was 0.1256, the likelihood of the data given the null was 6.6953, and the Bayes factor was 0.02. The Bayes factors were less than a third, so there was substantial evidence for nonsignificant differences of mood between the two conditions, indicating that the differences in approach tendency between the two conditions were not due to emotion.

Taking all of our results together, we did not find the hypothesized moderating effect of gender on the influence of the subjective feeling of the amount of money one possesses on individuals' tendency to approach the attractive alternative, suggesting that both men and women with relatively more money are more likely to choose the extra-pair mating strategy than those with less money. However, we did find that the men selected a closer seat to the attractive member of the opposite sex than the women in both the relatively wealthy and relatively poor conditions. This result is consistent with previous findings that committed women are more likely to distance themselves from the opposite sex than men (Lydon et al., 2008).

## General discussion

In the current studies, we used money-priming strategies to create the feeling of having relatively more or less money and examined how this feeling influences individuals' mating strategies. Our results showed that the feeling of having relatively more money caused the men, but not the women, to feel less satisfied with their partners' physical appearance and led both the men and women to approach an attractive member of the opposite sex more closely than if they felt they had relatively less money. Generally speaking, these findings are consistent with the evolutionary proposition that individuals adopt conditional mating strategies in response to environmental conditions such as resource cues (Gangestad and Simpson, 2000). Differences in the amount of money possessed cause significant variation in mating strategies within each gender. For men, the within-sex differences derive from the difference in their perceived mate value. For women, access to money might induce different reproductive benefit-cost analyses and the variance in the relative importance of a mate's good genes over parental investment. In other words, in ancient times, both men and women might tend to make an adaptive trade-off to maximize their reproductive benefits.

Interestingly, we did not find that women would make higher demands regarding men's physical appearance when they were primed to feel relatively wealthy. One possible reason for this is that individuals' mate preferences could be conditional on their self-perceived mate value. Furthermore, self-perceived mate value is sex specific. Men's mate value is based more on resources than women's mate value, while women's mate value depends more on physical attractiveness than men's mate value. Therefore, the difference in self-perceived resources generates the difference in men's partners' satisfaction with their partners' physical appearance, while for women, the effect is much smaller. An alternative explanation could be that possession of money plays a significant role in men's intrasexual competition, whereas women may experience less sexual selection pressure and have less need for intrasexual competition than men. Thus, compare to men, the effect of money may be less relevant to women. Except that, the selectivity of sample could also contribute to this result. We asked a sample of committed individuals who were already in long-term relationships to give ratings on their current partner's characteristics. As mentioned earlier, for committed women, making higher demands regarding a current partner might lead to reproductive cost by impairing the stability of the relationships. Thus, relationship status could be a critical factor that influences women's adaptive trade-off. Perhaps, for a similar reason, we did not find any effect of money on women's satisfaction with their partners' resources.

In Study 2, the finding that women chose a seat further away from the attractive member of the opposite sex's seat than the men did may reflect stable gender differences in mating strategies: men generally seek more partners than women to ensure reproductive success (Buss and Schmitt, 1993). Therefore, men are more likely to grasp every opportunity to approach an alternative mate and engage in extra-pair mating. However, it is noteworthy that the effects of money on the women's approach tendency toward a romantic alternative were not smaller than the effects on the men's approach tendency. This finding is inconsistent with our hypothesis of the smaller effects for women than for men in this situation because women are stronger protectors of romantic relationships (Lydon et al., 2008).

The behavioral measure used in Study 2 could have contributed to the nonsignificant gender difference in the tendency to approach the attractive alternative under the influence of money. The self-report results could be biased by social desirability concerns or limitations of self-knowledge. Previous studies have provided evidence that there is discrepancy between self-report and actual choices in mate selection preferences and have suggested drawing conclusions based on self-reported data with caution (Todd et al., 2007). In the current study, participants were blind to the purpose of the experiment. Being unaware of what was being measured, the women may have failed to hide their attitudes or inhibit their interest in the attractive alternative. In other words, we observed their actual behaviors (instead of self-reports) to prevent social desirability or self-knowledge from biasing their responses. This may suggest that women's insistence on loyalty is largely influenced by external norms related to gender roles.

Taken together, our findings show that both men and women use mixed mating strategies under different money-priming conditions. These findings suggest that money does have the potential to influence romantic relationships. Individuals' satisfaction with a current partner and their interest in a romantic alternative are significantly influenced by the amount of money they possess. This suggests that money could be one of the important factors in determining the stability of romantic relationships. Our findings also imply that the social distancing effect of money found in prior studies (e.g., Vohs et al., 2006; Mogilner, 2010) might not occur in the mating context. In the situation with an attractive alternative, money may exert a social engagement effect on both men and women.

## Limitations and directions for future research

Despite the interesting causal effects found in our two experiments, the current research has limitations. First, participants in the two studies were college students in dating relationships. Compared with married couples, dating relationships are generally less committed and less stable. Our findings thus cannot be directly generalized to marital relationships. Future studies should sample married individuals and examine if the money-priming effects can still be found. Second, and more importantly, we did not explore the psychological processes that mediate the influences of money on mating strategies. Future studies should examine whether men's perception of their own worth underlies the effects of money on their satisfaction with their partners and identify the mediators that underlie individuals' approach behaviors toward an attractive alternative. Third, in Study 2, we failed to find sex differences in the effects of money on individuals' tendency to approach an attractive alternative. Findings from this study are not enough to testify whether the behavioral measure is the reason why women's short-term mating decisions are significantly affected by resources. Future studies should examine this possibility by comparing the results of behavioral measures and self-reports in similar situations; this would give us a better understanding of how to access individuals' mating choices and allow us to understand these evolutionary mechanisms more accurately. Fourth, we did not include another factor that women desire for in a long-term mate—“good father,” which consists of a set of male attributes such as kindness and love and is closely related to direct paternal care (Lu et al., 2015). In Study 1, we focused on mate preferences for physical attractiveness and resources because significant gender differences have consistently been found in the two attributes. However, “kindness” was actually rated higher than “attractiveness” and “earning capacity” for both men and women in previous mate preference investigations (Buss, 1989; Chang et al., 2011b). Moreover, Lu et al.'s (2015) research reveals that women prefer “good father” over “good gene” and “good provision” under good economic conditions. Therefore, future studies should look into potential differences in mate preference for “good father” under different economic conditions. Lastly, we did not use the original Ideal Partner Scales in Study 1. We shortened the scale due to the fast and short-lived priming effect (Hermans et al., 2001). We also modified some items in order to make them more suitable for our Chinese student samples. Although the reliability coefficients of the revised scales were found to be acceptable, the psychometric properties of such shortened scales should be further examined in future studies.

## Conclusions

Given its ubiquitous presence in daily life, money has been found to exert a significant impact on our romantic relationships. The current studies focused on mating strategies and explored how money induces individuals' mating decisions in long-term and extra-term contexts under the framework of evolutionary psychology. Findings from our two experiments reveal that the feeling of having relatively more or less money could cause differences in mating strategies, implying that people may adjust their strategies to environmental conditions. From the perspective of evolution, these conditional mating strategies serve as solutions to the adaptive problems our ancestors faced in ancient times. These psychological mechanisms still play important roles in human mating. The practical implication of our findings is to remind people to pay attention to the potential changes brought about by changes in the amount of money they possess. In the discussion about relationship problems and solutions, the influence of money could be considered seriously. There is no harm in being vigilant when great changes take place in family or societal economics.

## Author contributions

Both YL and JL engaged in the design of the research, data collection and analysis, and drafting and revising the work. DC also participated in the design of the work and data analysis, and revised the paper very critically. BZ participated in the discussion of the experiments, data collection and paper revision. All the authors have approved of the version's publishment and agreed to be accountable for all aspects of the work.

## Acknowledgments

This research was supported by Beijing Higher Education Young Elite Teacher Project (YETP0247).

### Conflict of interest statement

The authors declare that the research was conducted in the absence of any commercial or financial relationships that could be construed as a potential conflict of interest.
